# Neoadjuvant docetaxel for operable breast cancer induces a high pathological response and breast-conservation rate

**DOI:** 10.1038/sj.bjc.6600916

**Published:** 2003-04-29

**Authors:** S Amat, P Bougnoux, F Penault-Llorca, F Fétissof, H Curé, F Kwiatkowski, J-L Achard, G Body, J Dauplat, P Chollet

**Affiliations:** 1 Centre Jean Perrin, 58 rue Montalembert, 63011 Clermont-Ferrand Cedex 1, France; 2 INSERM U484, rue Montalembert, 63005 Clermont-Ferrand Cedex, France; 3 CHU–Hôpital Bretonneau, 2 Boulevard Tonnelé, 37 044 Tours Cedex 1, France

**Keywords:** breast, docetaxel, induction, neoadjuvant, taxane

## Abstract

Docetaxel (Taxotere®), alone or in combination with other anticancer agents, has proven efficacy in the first- and second-line treatment of metastatic breast cancer. This phase II study investigated the efficacy and tolerability of docetaxel as neoadjuvant chemotherapy in women with stage II–III primary operable breast cancer. Patients (*n*=88) were treated with six cycles of docetaxel at 100 mg m^−2^ every 21 days, followed by definitive surgery and radiotherapy. After six cycles of docetaxel, the overall clinical response rate was 68.4% (CI 95%: 58.1–78.7%), including 19.0% complete remissions. Breast conservation was achieved in 72.4% of patients. A high pathological complete response (pCR) rate in breast was confirmed in 15 patients (19.8% (CI 95%: 10.8–28.8%)) on Chevallier's classification restricted to breast and in 27 patients (35.5% (CI 95%: 24.7–46.3%)) on Sataloff's classification. After a median follow-up of 30.8 months, 19 recurrences were documented with a median time to first recurrence of 17.3 months. Patients with stage III tumours had more recurrences than patients with stage II tumours (*P*=0.02). The principal toxicity of docetaxel is myelosuppression and 70.5% of patients developed grade III or IV neutropenia with 13.6% developing neutropenic sepsis. There was no case of severe cardiac toxicity, thrombocytopenia or any other serious adverse events. In conclusion, neoadjuvant docetaxel induces a high pCR and breast-conservation rate. Docetaxel monotherapy is a highly effective regimen that merits formal comparison with currently used combination regimens in a randomised phase III study.

The goal of chemotherapy given in the adjuvant or neoadjuvant (preoperative) setting is to eradicate occult distant metastases and to increase ultimately the probability of disease-free survival (Bear, 1998; Gradishar, 1999). Systemic therapy has been used for many years as a primary treatment in locally advanced or inflammatory breast cancer. More recently, the value of neoadjuvant chemotherapy has been recognised and extended to operable breast cancer for tumours that are too large to be treated immediately by conservative surgery. The aim of neoadjuvant chemotherapy is to reduce tumour size before surgery, and hopefully result in a lower rate of total mastectomy. Breast-conservation therapy can be performed in >50% of all cases, despite a nonnegligible rate of local recurrences (23.1 *vs* 8.7% for patients treated by adjuvant chemotherapy), without compromising patients' outcome (Mauriac *et al*, 1991). The quality of patients' response to this strategy, which is usually followed by locoregional therapy (surgery and/or radiotherapy), seems to be a prognostic factor in many studies (Scholl *et al*, 1991; Calais *et al*, 1994; Bonadonna *et al*, 1998; Brenin and Morrow, 1998; Ferriere *et al*, 1998). This has become a common approach for the treatment of a variety of neoplasms, including oesophageal, testicular, lung and breast cancers, osteosarcoma and squamous cell cancers of the head and neck.

Neoadjuvant chemotherapy in patients with operable breast cancer needs to be evaluated in prospective clinical trials before it is firmly established as the standard clinical practice (Mamounas, 1998). Furthermore, information on the differential histological responses of primary breast tumours and axillary metastases to neoadjuvant chemotherapy is limited (Chevallier *et al*, 1993; Sataloff *et al*, 1995; Chollet *et al*, 1997). Currently, only a small number of studies have published data concerning the number and outcome of patients with a complete pathological response of both the primary tumour and axillary lymph nodes after neoadjuvant chemotherapy (Feldman *et al*, 1986; Fisher and Mamounas, 1995, Fisher and Mamounas, 1997; Kuerer *et al*, 1999).

Docetaxel has been shown to be one of the most active cytotoxic agents. It is a promising candidate for new therapeutic strategies in patients with breast cancer, and is therefore potentially useful for neoadjuvant chemotherapy (Cortes and Pazdur, 1995; O'Leary *et al*, 1998; Costa *et al*, 1999). As a single agent, docetaxel has shown marked clinical activity in the treatment of anthracycline-resistant and chemotherapy-naïve (for metastatic disease) breast cancer, achieving response rates of 34 and 50–72%, respectively (Ravdin *et al*, 1995; Valero *et al*, 1995; Marty *et al*, 1997; Valero, 1997). In a randomised phase III trial in first-line metastatic breast cancer docetaxel (100 mg m^−2^) exhibited superior efficacy and tolerability compared with doxorubicin (at the optimal dose of 75 mg m^−2^) (Chan *et al*, 1999). The role of docetaxel (both as a single agent and in combination) in early or locally advanced breast cancer is currently being investigated (Gradishar, 1997; Mamounas, 1998; Costa *et al*, 1999). These clinical studies are examining the biological determinants of response and potential resistance to docetaxel.

This phase II trial was performed to assess the efficacy and safety of docetaxel as neoadjuvant chemotherapy in patients with operable stage II–III breast cancer. This involved evaluating response rates, and more specifically the pathological response rate.

## PATIENTS AND METHODS

In this phase II trial, neoadjuvant chemotherapy was administered to patients with operable nonmetastatic breast carcinoma. The primary objective was to assess the pathological response in the breast and axilla after six infusion cycles of docetaxel. However, any patient who failed to show evidence of a partial response after four cycles did not receive the two subsequent infusion cycles.

The eligibility criteria for this study were as follows: age <70 years, histologically proven invasive carcinoma, no metastatic spread, no prior specific treatment, no prior history of heart disease, adequate biological functions, World Health Organization (WHO) performance status of 0 or 1 and written informed consent. The study was conducted in accordance with the Hong Kong Amendment of the Declaration of Helsinki and approved by the Ethics Committee of Auvergne before commencement. The baseline workup included a complete history and physical examination, complete blood cell count, blood chemistry analysis, tumour markers, electrocardiography, chest X-ray, bone scan and liver ultrasound. Initial staging comprised a complete clinical examination, mammography and ultrasound; histological diagnosis was mandatory for the primary tumour and nodes.

Two centres were involved in the recruitment and treatment of patients. The method of histological diagnosis varied depending on the centre: needle core biopsy and per cutaneous cytology of palpable lymph node (Group 1), and surgical biopsy and axillary dissection (Group 2).

### Neoadjuvant chemotherapy

Oral methylprednisolone (48 mg) was administered 12, 3 and 1 h before, and 12, 24 and 36 h after each cycle of neoadjuvant chemotherapy. The regimen of docetaxel used was 100 mg m^−2^ given intravenously over 1 h, every 21 days for a total of six cycles.

Blood counts were performed once a week every week during the 21-day interval. Dose reductions and cycle delay were planned in case of severe haematological and nonhaematological toxicities. Subsequent treatments were administered 21 days after the previous treatment only if the patient's neutrophil count was ⩾1500 mm^−3^ and the platelet count was ⩾100 000 mm^−3^. If either count was below these levels, treatment was delayed for 1 week and in case of nonrecovery or nonhaematological toxicity ⩾grade 2 at day 21, the dose of docetaxel given at the next cycle was reduced by 25%.

### Locoregional treatment

Surgery consisted of lumpectomy if the residual tumour size was <3 cm, or modified radical mastectomy if the tumour size was ⩾3 cm and in cases of extended intracanalar disease. Surgical dissection of the axilla was performed after chemotherapy for Group 1 patients. Radiotherapy was given after surgery with a total dose of 50–60 Gy to the breast, internal mammary lymph nodes and supraclavicular/high axillary lymph nodes.

### Adjuvant therapy

When significant residual disease remained, adjuvant chemotherapy was considered on an individual basis and left to the clinician's judgement. Adjuvant hormonotherapy (tamoxifen) could be given for 2–5 years to postmenopausal patients presenting oestrogen-receptor-positive tumours.

### Assessment of response

Clinical, mammographic and ultrasound measurements were recorded before treatment and every two cycles during neoadjuvant chemotherapy. Clinical responses were evaluated by the decrease in tumour and node volumes (the product of the two greatest perpendicular dimensions), and were calculated according to the WHO recommendations (Miller *et al*, 1981). A global clinical response was estimated using the echographic response in the majority of cases, which accurately reflects the pathological response (Ferriere *et al*, 1998). In the other cases, the global clinical response corresponded to the worst of the responses obtained by the two other methods of measurement if echographic response was not available. The pathological response was independently evaluated after surgical resection of the remaining tumour, and then finally reviewed by an independent pathologist using the Chevallier *et al* (1993) and Sataloff *et al* (1995) classifications. As pathological response in axillary nodes could not be determined in Group 2, Chevallier's classification was modified to evaluate response in breast only. This modification allowed an accurate comparison between the two groups. Pathological response was then evaluated as follows:

- *Class* 1: disappearance of all tumour either on macroscopic or microscopic assessment;
- *Class* 2: presence of *in situ* carcinoma;
- *Class* 3: presence of invasive carcinoma with stromal alteration, such as sclerosis or fibrosis;
- *Class* 4: no or few modifications of the tumoral appearance.

### Statistical methods

Statistical analysis was descriptive. The comparisons of the different clinicopathological characteristics, response rates or other parameters between the two groups gave rise to an exploratory analysis with a significant *P*-value of 0.05.

## RESULTS

From September 1997 to November 2000, 88 patients entered this phase II trial. The patients' median age was 46 years (range, 29–66 years), and 49 women (55.7%) were premenopausal. In all, 86 tumours were ⩾3 cm in diameter and two tumours were <3 cm, but located in the central area of the nipple, with a lymph node status of N0 or N1. The median largest diameter of the primary tumour was 50 mm (range, 20–130 mm). Further, tumour characteristics are shown in Table 1

**Table 1:** Tumour characteristics

	**No. of patients (%)**			
**Characteristic**	**Overall (*n*=88)**	**Group 1 (*n*=51)**	**Group 2 (*n*=37)**	***P*-value**
T				
T2	53 (60.2)	32 (62.8)	21 (56.8)	
T3	28 (31.8)	16 (31.4)	12 (32.4)	0.68
T4^a^	7 (8.0)	3 (5.8)	4 (10.8)	

N				
N0	44 (50.0)	27 (52.9)	17 (46.0)	0.52
N1	44 (50.0)	24 (47.1)	20 (54.0)	

Pathology				
Invasive ductal	71 (80.7)	42 (82.4)	29 (78.4)	
Invasive lobular	12 (13.6)	5 (9.8)	7 (18.9)	0.31
Unspecified invasive carcinoma	5 (5.7)	4 (7.8)	1 (2.7)	

Scarff–Bloom–Richardson grading				
I	2 (2.3)	1 (2.0)	1 (2.7)	
II	39 (44.3)	23 (45.1)	16 (43.2)	0.83
III	42 (47.7)	22 (43.1)	20 (54.1)	
Not graded	5 (5.7)	5 (9.8)	0 (0.0)	

Hormonal receptors				
ER− PgR−	28 (31.8)	17 (33.3)	11 (29.8)	
ER− PgR+	3 (3.4)	2 (3.9)	1 (2.7)	
ER+ PgR+	39 (44.4)	19 (37.3)	20 (54.0)	0.77
ER+ PgR−	9 (10.2)	5 (9.8)	4 (10.8)	
Not classified	9 (10.2)	8 (15.7)	1 (2.7)	

Cell kinetics				
Presence of an aneuploid population	44 (50.0)	26 (51.0)	18 (48.6)	0.36
Not performed	28 (31.8)	18 (35.3)	10 (27.0)	
S-phase ⩾5%	32 (36.4)	24 (47.1)	8 (21.6)	0.28
Not performed	44 (50.0)	20 (39.2)	24 (64.9)	

a Some patients with T4 tumours entered this phase II trial. These comprised T4a and T4b tumours and not inflammatory carcinoma. Consequently, it was considered as a minor protocol violation and these patients were included in the analysis.

ER, oestrogen receptor; PgR, progesterone receptor.

. In all, 44 patients (50.0%) did not have clinically detectable lymph node involvement at diagnosis, 61 patients (69.3%) had stage II disease and 27 patients (30.7%) had stage III disease (20 stage IIIa and seven stage IIIb). In addition, all patients had at least one other adverse prognostic factor: clinical lymph involvement (44 N1); high Scarff–Bloom–Richardson (SBR) grading (42 grade III); aneuploidy (44 cases); and negative oestrogen and progesterone receptors (28 cases).

At the initial staging, 51 patients underwent needle core biopsy and node cytology (Group 1), and 37 had surgical biopsy and axillary dissection (Group 2). As shown in Table 1, the clinical and pathological characteristics of the two groups were similar. In Group 2, the median volume of surgical primary biopsy, while not recorded, has been estimated at 10–20% of the total initial tumour volume. The median number of nodes collected was 13 (range, 7–27) and the median number of metastatic nodes was five (range, 0–22).

### Treatment management

A median number of six cycles (range, 1–6) of neoadjuvant docetaxel chemotherapy were administered, the median dose being 100 mg m^−2^ (range, 82–106 mg m^−2^) in 88 patients. One patient stopped treatment after five cycles because of septicaemia. The treatment was also stopped for two patients because of lack of response at four cycles, as planned by the protocol. However, 24 patients (23 with minor response and one with no change) were given the two subsequent infusion cycles. Two patients were considered as noneligible: one patient because of lung metastases and another because of predominant *in situ* carcinoma observed at final review. Of the 86 eligible patients, six were withdrawn from the study for the following reasons: allergy to docetaxel (*n*=3), lack of response after four cycles (*n*=2) and toxicity (*n*=1). Thus, 80 patients were included in the per protocol analysis of clinical response.

In all, 10 patients did not undergo surgery: four because of disease progression, one because of lack of response after four cycles, three who were withdrawn from the study (because of drug allergy) and two who refused surgery (because of a complete clinical response to docetaxel treatment). Thus, 76 of the 86 eligible patients underwent surgery (42 in Group 1 and 34 in Group 2) and were evaluable for pathological response. Breast-conserving surgery was performed in 55 patients (72.4%) and a modified radical mastectomy in 21 patients (10 for residual tumour and 11 for *in situ* carcinoma only). There was no difference between the two groups in terms of surgical treatment (*P*=0.20).

After surgery, all 76 patients received radiotherapy. Subsequently, patients received adjuvant chemotherapy at investigator discretion (*n*=37; 48.7%) and/or hormonotherapy with tamoxifen (*n*=34; 44.7%). The adjuvant chemotherapy, which consisted of six cycles of the FVC or FEC regimens (5-fluorouracil/vinorelbine or epirubicin/cyclophosphamide), was given to 15 out of 42 patients in Group 1 and to 22 out of 34 patients in Group 2.

### Response

As shown in Table 2

**Table 2:** Clinical response rate to neoadjuvant treatment

	**No. of patients (%)**			
	**Clinical response**	**Mammographic response**	**Echographic response**	**Global clinical response**
Overall (*n*=80)				
Complete response (CR)	27 (36.0)	18 (45.0)	11 (17.5)	15 (19.0)
Partial response (PR)	28 (37.3)	10 (25.0)	31 (49.2)	39 (49.4)
Minor response/no change	11 (14.7)	5 (12.5)	11 (17.5)	17 (21.5)
Progression	9 (12.0)	7 (17.5)	10 (15.8)	8 (10.1)
** ORR (CR+PR) No. (%; 95 CI)**	**55 (73.3; 63.3–83.3)**	**28 (70.0; 55.8–84.2)**	**42 (66.7; 55.1–78.3)**	**54 (68.4; 58.1–78.7)**

Group 1 (*n*=45)				
** ORR (CR+PR) No. (%; 95 CI)**	**34 (77.2; 64.8–89.6)**	**18 (66.7; 48.9–84.5)**	**27 (75.0; 60.9–89.1)**	**34 (75.6; 63.1–88.1)**

Group 2 (*n*=35)				
**ORR (CR+PR) No. (%; 95 CI)**	**21 (67.8; 51.4–84.2)**	**10 (77.0; 45.0–94.7)**	**15 (55.5; 36.8–74.2)**	**20 (58.9; 42.4–75.4)**

ORR: overall response rate.

, response rates (for the per protocol population) obtained after neoadjuvant docetaxel therapy were evaluated by clinical, mammographic and ultrasound measurements. The global clinical response corresponded to the echographic response in the majority of cases or to the worst of the responses obtained by the two other methods of measurement if echographic response was not available. Neoadjuvant docetaxel therapy resulted in an overall clinical response rate of 73.3%, including 36.0% complete remissions. Overall mammographic and echographic response rates were 70.0 and 66.7%, respectively. The overall global response rate was estimated to be 68.4%, with 19.0% complete responses. The overall global response rate for the intent-to-treat population (86 patients) was also calculated (62.8% [CI 95%: 52.6–73.0%], with 17.4% complete responses). There was no difference in clinical response between Groups 1 and 2, irrespective of the assessment method used (*P*<0.35) or the stage of disease (II *vs* III, *P*=0.96).

### Histopathological evaluation

As shown in Table 3

**Table 3:** Pathological response rate to neoadjuvant treatment

	**No. of patients (%)**		
**Pathological response**	**Overall (*n*=76)**	**Group 1 (*n*=42)**	**Group 2 (*n*=34)**
Restricted Chevallier's classification			
Class 1: no tumour in breast (pCR)	10 (13.2)	3 (7.1)	7 (20.6)
Class 2: *in situ* carcinoma only	5 (6.6)	2 (4.8)	3 (8.8)
Class 3: invasive carcinoma with alteration	41 (53.9)	26 (61.9)	15 (44.1)
Class 4: unmodified invasive carcinoma	20 (26.3)	11 (26.2)	9 (26.5)
**ORR (Classes 1+2) No. (%; 95% CI)**	**15 (19.8; 10.8–28.8)**	**5 (11.9; 4.1–25.9)**	**10 (29.4; 14.1–44.7)**

Sataloff's classification			
Class A: complete+quasicomplete response	27 (35.5)	13 (30.9)	14 (41.2)
Class B: partial response	11 (14.5)	6 (14.3)	5 (14.7)
Class C: minor response	18 (23.7)	12 (28.6)	6 (17.7)
Class D: no response	20 (26.3)	11 (26.2)	9 (26.4)
**ORR (Class A) No. (%; 95% CI)**	**27 (35.5; 24.7–46.3)**	**13 (30.9; 16.9–44.9)**	**14 (41.2; 24.7–57.7)**

ORR: overall response rate.

, the overall pathological complete response (pCR) rate was 19.8%, according to Chevallier's classification restricted to breast. For patients who had a pCR, the median tumour size before chemotherapy was 50 mm (range, 20–90 mm). Among the patients having a global complete clinical response prior to surgery, six of 13 operated patients (46.2%) were shown to have pathological evidence of a residual tumour. Conversely, among the patients showing an incomplete clinical response (partial or minor response, no change or progression), eight out of 60 operated patients (13.3%) were classified as pCR according to Chevallier's classification restricted to breast. The overall pCR rate was 35.5% according to Sataloff's classification. For patients classed as pCR after docetaxel, median tumour size before chemotherapy was 50 mm (range, 20–90 mm). When a global complete clinical response was obtained before surgery, a residual pathological tumour was found in 23.1% of these patients (three out of 13). Among the patients showing incomplete clinical response, 15 out of 60 (25.0%) had a pCR according to Sataloff's classification. Any difference observed between Groups 1 and 2 was not statistically significant (*P*=0.06 and 0.35 according to the restricted Chevallier's and Sataloff's classifications, respectively). Similarly, there was no difference in pathological response, irrespective of the stage of disease (II *vs* III, *P*=0.64 and 0.43 according to restricted Chevallier's and Sataloff's classifications, respectively).

### Follow-up

On 3 July 2001, the median follow-up period was 30.8 months (range, 8.2–45.5 months). In all, 19 recurrences were observed (split evenly between Groups 1 and 2), with the median time to first recurrence being 17.3 months (range, 7.6–24.2 months). Eight patients (9.3%) have died. In all, 19 recurrences were observed:

- one local in the breast;
- six both local and metastatic (two in lung, two in bone, one in multiple site and one in cervix nodes);
- and 12 with distant metastases (three in multiple sites, three in bone, two in lung, two in brain, one in liver and one in uterus).

Of these 19 patients with recurrence, following neoadjuvant chemotherapy, nine (47.7%) had shown an overall global response (two complete and seven partial responses). Patients with stage III disease presented more recurrences than patients with stage II disease (38 *vs* 15%, *P*=0.02). Of the 16 operated patients who presented recurrences, three (18.8%) had pCR according to the restricted Chevallier's classification and seven (43.8%) according to Sataloff's classification.

### Tolerability of neoadjuvant chemotherapy

From a total of 431 infusion cycles evaluated, the associated extrahaematological toxicities are shown in Table 4A

**Table 4:** (A) Extrahaematological^a^ and (B) haematological^b^ toxicities of neoadjuvant treatment

	**No. of patients by WHO grade (%)**					
**Event**	**I**	**II**	**III**	**IV**	**Total no. of patients (%)**	**No. of events (% of cycles)**
(A)						
Acute hypersensitivity reactions	0 (0)	39 (44.3)	0. (0)	0 (0)	39 (44.3)	73 (15.1)
Gastrointestinal toxicity	0 (0)	43 (48.9)	0. (0)	0 (0)	43 (48.9)	76 (15.7)
Asthenia	0 (0)	40 (45.5)	0. (0)	0 (0)	40 (45.5)	68 (14.0)
Myalgias and arthralgias	0 (0)	27 (30.7)	1. (1.1)^c^	0 (0)	28 (31.8)	50 (10.3)
Cutaneous toxicities	0 (0)	33 (37.5)	0.	0 (0)	33 (37.5)	44 (9.1)
Moderate oedema	0 (0)	28 (31.8)	0.	0 (0)	28 (31.8)	36 (7.4)
Paresthesias	0 (0)	13 (14.8)	1. (1.1)	0 (0)	14 (15.9)	24 (4.9)
Alopecia	6 (6.8)	16 (18.2)	38. (43.2)	4 (4.6)	64 (72.7)	302 (62.3)

(B)						
Neutropenia	2 (2.3)	7 (8.0)	19. (21.6)	43 (48.9)	71 (80.7)	244 (50.3)
Leucopenia	12 (13.6)	31 (35.2)	23. (26.1)	0 (0)	66 (75.0)	199 (41.0)
Anaemia	16 (18.2)	2 (2.3)	0. (0)	1 (1.1)	19 (21.6)	34 (7.0)
Thrombocytopenia	0 (0)	0 (0)	0. (0)	0 (0)	0 (0)	0 (0)

a In total, 89% of cycles were available for extrahaematological toxicities (431 cycles).

b As a result of some missing blood counts, 56% of cycles were available for leucopenia (262 cycles) and neutropenia (263 cycles), 57% for anaemia (266 cycles) and thrombocytopenia (265 cycles).

c The dose of docetaxel given was reduced by 10% until the end of treatment for one patient who presented with severe myalgias.

. Alopecia was nearly universal, but reversible, except in four patients who presented with grade IV. No severe cardiac toxicity occurred despite nine cases of tachycardia with dyspnoea. There were no significant changes in renal or hepatic parameters. Treatment was stopped in only three patients who suffered acute allergy to docetaxel at the beginning of their second infusion.

Table 4B details the haematological toxicities of neoadjuvant treatment. In all, 12 patients (13.6%) developed febrile neutropenia (i.e. neutropenia grade IV and fever grade II) which was treated by antibiotic therapy for a median duration of 7 days (range, 0–10 days). The median duration of a febrile episode was 2 days (range, 2–8 days), and four patients required hospitalisation. Two Group 2 patients with significant haematological toxicity (grade IV febrile neutropenia) after the second and third cycle were given haematopoietic growth factor; although no dose reduction or delay in treatment was required. Grade III–IV neutropenia was observed in 62 patients (70.5%). In all, 23 patients (26.1%) developed grade III leucopenia; no patient developed grade IV leucopenia. One patient (1.1%) with grade IV anaemia required a blood transfusion, and the dose of docetaxel was reduced by 25% until the end of treatment. In all, 18 patients (20.5%) developed grade I–II anaemia. There was no thrombocytopenia, and no other significant adverse event was observed.

## DISCUSSION

The aim of the present phase II study was to assess the efficacy and tolerability of six cycles of docetaxel at 100 mg m^−2^ every 21 days as neoadjuvant chemotherapy in patients with operable breast cancer.

Although it is perhaps too early to draw definitive conclusions about the outcome of these patients, neoadjuvant docetaxel produced very effective tumour response and was well tolerated. Neoadjuvant docetaxel also offered the possibility of breast conservation in 72.4% of patients who otherwise would have undergone a mastectomy. The global clinical response rate (68.4%) and the pCR rate (19.8 and 35.5% according to the restricted Chevallier's and Sataloff's classifications, respectively) were very high for monotherapy. They are comparable with the results of Estévez *et al* (2000), who investigated the response rate of weekly docetaxel 40 mg m^−2^ administered for a 12-week period in 54 patients with stage II–III breast cancer. In their preliminary results, this regimen demonstrated an overall clinical response rate of 71.0% (complete response, 26.3%; partial response, 44.7%) and a pCR rate of 21%.

In our study, tumour reduction occurred progressively, with only 40% of the complete responses having been obtained after four cycles. After six cycles, our results suggested that this longer period of monotherapy ensured a good response rate, and in particular a high pathological response rate. This fact, taken together with our previous findings (Cure *et al*, 1997; Ferriere *et al*, 1998), confirm that a longer period of neoadjuvant chemotherapy seems to improve the complete response rate, and possibly the rate of breast conservation. Furthermore, Swain *et al* (1987) have reported that the median number of cycles required to reach a partial or complete response was three and five, respectively.

We noted that the rates of response between the two groups differed according to the classification system used (11.9 *vs* 29.4% in Groups 1 and 2 with the restricted Chevallier's classification compared with 30.9 *vs* 41.2% in Groups 1 and 2 with Sataloff's classification), but not according to the stage disease. This gives prominence to the importance of pathological response evaluation, particularly in terms of patients' outcome. In the literature, there was a small, insignificant advantage in disease-free survival for patients reaching a pCR after neoadjuvant chemotherapy (Kuerer *et al*, 1999). In the present study, of the 16 operated patients who presented recurrences, three (18.8%) had pCR according to the restricted Chevallier's classification and seven (43.8%) according to Sataloff's classification. Moreover, patients with stage III disease presented more recurrences than patients with stage II disease (38 *vs* 15%, *P*=0.02), as previously described (Jacquillat *et al*, 1990; Kato *et al*, 2001; Menard *et al*, 2002). A total of 49% of patients received adjuvant chemotherapy and 45% hormonotherapy. Intensification of the adjuvant treatment with a second noncrossresistant regimen or high-dose chemotherapy with peripheral blood stem cells support may be an advantage for patient outcome. Conversely, for patients who presented a pCR, the identification of factors predicting the response to treatment and patient outcome may assist the clinician to more accurately select patients who may or may not benefit from such a strategy. However, a longer follow-up period is needed to confirm these results.

Six cycles of docetaxel 100 mg m^−2^ was safe, with the main haematological toxicity being neutropenia. As only 56% of cycles were assessable for haematological toxicity, the rate of significant neutropenia could be underestimated. Therefore, haematopoietic growth factors could be used to improve tolerance and avoid hospitalisation as much as possible. However, no dose reduction or delay in treatment was performed, and blood counts returned to normal before the next cycle. No severe extrahaematological toxicities (WHO grade ⩾3) were observed and the clinical tolerance was found to be comparable to the usual experience with docetaxel at this dose (Fumoleau *et al*, 1995; Chevallier *et al*, 1995).

Several studies have compared preoperative with postoperative chemotherapy in patients with operable breast cancer (Mauriac *et al*, 1991; Scholl *et al*, 1994, Scholl *et al*, 1995; Powles *et al*, 1995; Mamounas, 1998). The principal aim of these trials was to determine whether preoperative chemotherapy more effectively prolongs disease-free and overall survival than the same chemotherapy given postoperatively. Neoadjuvant therapy seemed to show better results in terms of rate of response to treatment and a reduction in the requirement for mastectomy. However, the results for survival rates are conflicting. Scholl *et al* (1994) initially observed a statistically significant difference in survival in favour of the neoadjuvant chemotherapy group, but they reported later that this advantage became insignificant (Scholl *et al*, 1995). Mauriac *et al* (1991) concluded that survival rates were identical in their two treatment groups. A study by Powles *et al* (1995) confirmed previous reports of a high rate of response to neoadjuvant therapy, but the follow-up period was too brief to evaluate the relapse rate or survival duration. The National Surgical Adjuvant Breast Project (NSABP) study B-18 demonstrated that in 1523 patients with operable breast cancer, preoperative chemotherapy resulted in high rates of clinical response, axillary nodal downstaging and increased rates of breast preservation (Mamounas, 1998). A low rate of pCR was found, but it was calculated from patients who had a complete clinical response and may not, therefore, be representative of all the patients who received preoperative chemotherapy. The authors concluded that there were no differences in progression-free or overall survival between the two groups. Hence, even if these studies showed that neoadjuvant chemotherapy did not demonstrate a greater survival, this strategy offers the possibility of downstaging and the avoidance of mastectomy in 75–80% of bulky tumours.

With the development of new taxanes, and the demonstration of the significant antitumour activity in patients with advanced breast cancer (Ravdin *et al*, 1995; Valero *et al*, 1995; Marty *et al*, 1997; Valero, 1997), clinical research is now focused on integrating docetaxel into combination regimens and into neoadjuvant and adjuvant schedules for patients with operable breast cancer. The biological determinants of response and resistance to docetaxel are also being examined (Powles *et al*, 1995; O'Leary *et al*, 1998). A very important question is whether there is true synergy between two or more drugs that are used simultaneously or successively. Several large phase II and randomised phase III trials are currently evaluating docetaxel used in combination, and/or sequentially, in the preoperative setting (Gradishar, 1997, Gradishar, 1998; Mamounas, 1998; Costa *et al*, 1999).

In a study by Gradishar *et al* (1997), 43 stage III patients received four cycles of docetaxel 100 mg m^−2^ on a 3-weekly schedule, followed by surgery, then four cycles of adjuvant doxorubicin plus cyclophosphamide, and also tamoxifen if the tumour was hormone-receptor positive or the patient was aged >50 years. The overall clinical response rate was 78% (complete response, 14%; partial response, 64%). Of the six patients with complete clinical response, only one (2%) showed pCR. von Minckwitz *et al* (1999) treated 42 patients with stage III operable breast cancer with four cycles of docetaxel 75 mg m^−2^ plus doxorubicin 50 mg m^−2^. An overall response rate of 93% (complete response, 33%; partial response, 60%) and a pCR rate of 5% were observed. The same regimen was administered to nine patients with locally advanced breast cancer (LABC). Preliminary results showed an overall response rate of 100% (complete response, 11%; partial response, 89%) with one patient (11%) showing a partial pathological response and one patient (11%) showing a minor pathological response. The Aberdeen Breast Study group is conducting a phase III trial in patients with stage III or LABC using docetaxel in sequential fashion following an anthracycline-based cyclophosphamide/doxorubicin/vin cristin/prednisolone (CVAP) regimen (Smith *et al*, 2002). In patients with tumours unresponsive to CVAP, docetaxel induced a 55% clinical response and a 2% pCR rates. For patients with initially responsive tumours, the sequential use of docetaxel also gave better results (94% clinical response and 34% pCR rates) than further cycles of CVAP (66% clinical response and 16% pCR rates). The efficacy of docetaxel over CVAP seems to have been demonstrated (*P*=0.001 for clinical response and *P*=0.04 for pathological response).

In conclusion, the results of the present study show that neoadjuvant chemotherapy with docetaxel is both effective and well tolerated in patients with early-stage operable breast cancer. In addition, neoadjuvant chemotherapy with docetaxel may improve breast-conservation rates. Randomised phase III studies are required to confirm the benefits of neoadjuvant *vs* postoperative adjuvant chemotherapy.
